# The African Medicines Agency - A potential gamechanger that requires strategic focus

**DOI:** 10.1371/journal.pgph.0004276

**Published:** 2025-02-14

**Authors:** Niteen Wairagkar, Benjamin Djoudalbaye, Inas Moubarak, Moustapha Zakari, Douglas N. Shaffer, Alex Juma Ismail, Dalia M.N. Abouhussein, Moji Christianah Adeyeye, Delese Mimi Darko, Adam Mitangu Fimbo, Richard T. Rukwata, Boitumelo Semete-Makokotlela, Emile Bienvenu, Julio Rakotonirina, Michele Sidibe, Jerome H. Kim, Minata Cessouma Samate, Nicaise Ndembi

**Affiliations:** 1 Vaccines for All, Pune, India; 2 African Medicines Agency Secretariat, Kigali, Rwanda; 3 International Vaccine Institute, Kigali, Rwanda; 4 African Medicines Harmonization Initiative, AUDA NEPAD, South Africa; 5 Egyptian Drug Authority (EDA), Agouza, Giza, Egypt; 6 NAFDAC, Zone 7, Abuja, Nigeria; 7 Ghana Food and Drugs Authority, Accra, Ghana; 8 Food and Drugs Authority, Tanzania; 9 Medicines Control Authority of Zimbabwe, Harare, Zimbabwe; 10 South African Health Products Regulatory Authority, Pretoria, South Africa; 11 Rwanda Food and Drugs Authority, Kigali, Rwanda; 12 African Medicines Agency, Kigali, Rwanda; 13 College of Natural Sciences, Seoul National University, Seoul, South Korea; 14 Africa Centres for Disease Control and Prevention (Africa CDC), Addis Ababa, Ethiopia; 15 Institute of Human Virology (IHV), University of Maryland School of Medicine, Baltimore, Maryland, United States of America; PLOS: Public Library of Science, UNITED STATES OF AMERICA

Regulatory agencies are critical to assure quality, safety, and efficacy or performance of medical countermeasures, including diagnostics, therapeutics, vaccines and health technologies. Initiatives that harmonize regulatory processes can help overcome bottlenecks and facilitate access to lifesaving tools. When a transnational regulatory agency is created, particularly on a continent like Africa, the complexities of each stakeholder’s triaging roles and responsibilities are enhanced multifold. This article presents a contextual analysis that could help enhance the African Medicines Agency’s (AMA) potential role with a clearer strategic focus.

The AMA was established by the African Union (AU) in 2019 as an independent regulatory institution overseeing approval and access to lifesaving products for the African population. The AMA treaty [1] provides a legal basis for establishment and the founding philosophy of this new regulatory body.

The African regulatory system is a complex, three-tier system of regulatory agencies [2] with 54 National Regulatory Agencies (NRAs) established at the national level. The World Health Organization (WHO) assesses the capacity of each NRA with a tool called WHO Global Bench-marking Tool (GBT)^3^ and categorizes the NRA with a maturity level (ML) ranking from 1-4, with ML4 being the highest-level, indicating capacity to regulate products with a high degree of stringency.

An ML-3 level defines a stable, well-functioning and integrated NRA that can assure the quality, safety and efficacy of products [3]. An approval by ML-3 NRA allows national manufacturers to apply for WHO pre-qualification and WHO Emergency Use Listing of their manufactured products [3]. As of December 2024, there are only eight countries with a ML-3 ranking on the African continent [4] and rest of the NRAs are at a maturity level of 1 or 2 indicating minimum capacity. This suggests that many African nations need to invest more in their national NRAs and elevate their standards to manufacture diagnostics, therapeutics, vaccines and other medical counter measure products.

To address some of the limitations of national-level capacity, there are 5 regional regulatory harmonization initiatives wherein countries within the region share their regulatory capacities through joint working protocols and procedures to approve the products for the region^2^. Though this facilitates quicker approval of products within each region, it is still difficult to ensure distribution of these products throughout the continent. At the continental level, African Medicines Regulatory Harmonization (AMRH) Initiative was established in 2009 [5] to harmonize regulatory requirements, establish standards and norms, and set up guidelines and joint procedures of working together for harmonized regulatory processes. It has been very effective technically and strategically, setting the stage for establishment of AMA but it lacked the political or regulatory status of an independent agency [2,5].

Product approval processes have evolved through this period and have differentiated regulatory pathways for approval during the peacetime for routine vaccines and therapeutics, and for emergency use authorizations during the emergencies set out by emerging pathogens. Initiatives like the Coalition for Epidemic Preparedness Innovations’ (CEPI) 100 Day Mission, set up and accepted by majority of countries after the COVID19 pandemic, seek to make diagnostics, therapeutics and vaccines available within 100 days of the declaration of Public Health Emergency of International Concern [6] (PHEIC). These initiatives set out expectations for the regulatory agencies to work within stringent timelines. The regulatory agencies will need a separate set of procedures, protocols, reliance mechanisms and guidance to meet these highly ambitious timelines during outbreaks and pandemics.

Though European Medicines Agency (EMA) [7] could be one model for AMA, there are clear Africa-spexific perspectives AMA needs to consider while setting out its own scope and mandate once operationalized. This requires complex set of strategies, pre-established procedures, protocols and pre-approved mechanisms to deal with peace times and emergency scenarios.

The Strengths, Weaknesses, Opportunities and Challenges (SWOC) analysis of AMA S1 Table indicates that though there are complexities and challenges for smooth working of AMA, there are opportunities for setting out a clearer longer-term scope of work and managing expectations of this nascent continental agency. We can set a course for success on the continent and become recognized as an equal among global regulatory leaders.

With this background, we offer some suggestions to enhance the potential value and impact of AMA:

1. AMA needs to be a politically, strategically, technically and financially independent agency at continental level. This independence, set out in the mandate, approved by the African Union and countries signing AMA treaty will make it truly continental technical agency.
2. AMRH has established several technical committees with representation from various regulators from within the continent, with procedures, protocols and guidance. These should be suitably modified, to be adopted and established as AMA standard and norms for harmonized regulation within Africa. This is an important function of AMA which will define its course and ensure sustainability.
3. Africa Centres for Disease Control and Prevention (Africa CDC) is another autonomous health institution of the Africa Union charged with the responsibility of prevention and control of diseases in Africa [8]. AMA and Africa CDC will need to work together during public health emergencies with pre-approved framework, guidance and protocols to quickly approve lifesaving products.
4. The AMA should create a platform to recommend and approve essential products during public health emergencies, ideally within 100 days of such emergencies.
5. There are several product categories (diagnostics, therapeutics, vaccines and biologicals, devices, etc.) which will be up for regulatory reviews and recommendations by AMA. With the current capacity and infrastructure, AMA may not initially be able to take care of all these product categories. AMA will need to assess the continental priorities and take up only the products where capacity is lacking at regional or national level.
6. AMRH Evaluation of Medicinal Product Technical Committee (EMP-TC) has established the guidance and listing of such continental prioritization [9] which is recommended to be adopted by AMA, e.g., vaccines and complex molecules should be dealt with at the continental level through AMA. Clear product categorization for regulatory reviews within a three-tier system will allow optimum utilization of existing capacities. AMA, Regional Harmonization Initiatives and NRAs need to work together to realize this ambitious scope of work.
7. WHO Prequalification [10] is the process of international standardization wherein certain categories of the products can be assessed by WHO for Programmatic Suitability for Prequalification (PSPQ) criteria. This WHO PQ is a requirement for the tendering process of international agencies like US PEPFAR, Global Fund for HIV, TB and Malaria, UNICEF and GAVI. Only few locally manufactured products from Africa have been WHO prequalified creating a barrier for access [2]. There is an expectation from the product development and regulatory ecosystem that the AMA, once operationalized, should take care of this issue at least for African Markets. Any product recommendation by AMA should be sufficient for procurement agencies to procure the products for at least African markets.

With these improvements, the AMA will play an indispensable role in regulating medical product development across Africa by promoting regulatory harmonization, supporting the development of local pharmaceutical and medical devices industries, strengthening regulatory capacity to improve access to high quality, safe, effective, and affordable medicines. Operationalizing AMA with clear strategic scope of work and priorities, will signal the new dawn of product approval/ recommendations that will support regional health security by paving the way to quicker access for populations that need them most.

## Supporting information

S1 Table Footnote below the table spell out all the acronyms. (DOCX)
