# Supplementary material for: The African Medicines Agency - A potential gamechanger that requires strategic focus
Source: PLOS Glob Public Health. 2025 Feb 14;5(2):e0004276. doi: 10.1371/journal.pgph.0004276 (PMC11828376; doi:10.1371/journal.pgph.0004276)
Supplement: S1 Table — (DOCX) [file pgph.0004276.s001.docx]

**S1 Table: SWOC analysis for African Medicines Agency**

| Strengths | Weaknesses | Opportunities | Challenges |
| --- | --- | --- | --- |
| AMA has Pan-African mandate and authority. Ratification of AMA treaty by a considerable number of AU member countries | Majority NRA yet to reach minimum WHO maturity level (ML3) for independent work. | Existence of regional economic communities, precedence and experience of joint assessment/ coordination and consortium of ML3 agencies, and mutual recognition for product registration, inspection and related activities. | Limited workforce and pool of experts in critical areas like emerging advanced therapeutic areas in GMP inspections and evaluations |
| Political will, convening power and support from Heads of State and Government. | Coordination of Research & Development activity across the value chain. | Existence of 8 ML3 national regulatory authorities | Limited local pharmaceutical and medical devices manufacturing. Many countries still rely on imports |
| Implementation of AU Agenda 2063 | Limited infrastructure for quality assessments | Establishment of technical and governance mechanism through Use of AMRH program | Limited access to technology platforms. |
| Availability of technical guidelines, standards and Joint continental procedures through decadal work of AMRH. | Three tier regulatory system will need extensive coordination. | Initiatives like Southern African Development Community (SADC) pooled procurement mechanism and  West Africa-Medicine Regulatory Harmonization pooled assessment and inspection mechanism | Utilization of TRIPS flexibilities |
| Ten technical committees established and functional to take care of AMA standards and norms | Absence of ratification (yet) by ALL regulators may create disparity between regulators. | Good working relationship with AUDA-NEPAD, Africa CDC, AfCTA, WHO and other development partners | National sovereignty issues undermining AMA’s impact |
| Willingness of international agencies to support regulatory harmonization through AMA in African continent | Limited resource (workforce, financial and legal) available. | Existence of WHO-prequalified laboratories across the continent  Existence of the AMRH Partnership Platform which coordinate all partner support on the continent | Large, continental NRA leader Member States pending ratification |
|  |  | Strong and growing clinical trials infrastructure experienced in conducting regulated clinical trials under GCP | National and regional complex Ethics Review Committee systems |

Africa CDC- Africa Centers for Disease Control and Prevention

AfCTA- The African Continental Free Trade Area

AMA- African Medicines Agency

AMRH- Africa Medicines Regulatory Harmonization Initiative

AU- African Union

AUDA-NEPAD – African Union Development Agency

GCP- Good Clinical Practices

GMP- Good Manufacturing Practices

ML3- Maturity Level 3

SADC- Southern African Development Community

TRIPS- Trade-Related Aspects of Intellectual Property Rights

WA-MRH - West Africa-Medicine Regulatory Harmonization

WHO - World Health Organization
